# Clinical features and outcomes of childhood interstitial lung disease: a tertiary center experience

**DOI:** 10.55730/1300-0144.6153

**Published:** 2026-01-05

**Authors:** Ayyüce ÜNLÜ, Şule Selin AKYAN SOYDAŞ, Satı ÖZKAN TABAKÇI, Işıl BİLGİÇ, Meltem KÜRTÜL ÇAKAR, Gamze AKCA DİNÇ, Hande YETİŞGİN, Çelebi YILDIRIM, Gökçen Dilşa TUĞCU, Dilber ADEMHAN TURAL, Sanem ERYILMAZ POLAT, Güzin CİNEL

**Affiliations:** 1 Division of Pediatric Pulmonology, Department of Pediatrics, Ankara Bilkent City Hospital, Ankara, Turkiye; 2 Division of Pediatric Pulmonology, Department of Pediatrics, Faculty of Medicine, Ankara Yıldırım Beyazıt University, Ankara, Turkiye

**Keywords:** Interstitial lung diseases, childhood, outcome, hypoxia

## Abstract

**Background/aim:**

Childhood interstitial lung diseases (chILD) constitute a rare and clinically complex group of disorders. This study aimed to characterize the clinical, radiological, and genetic features, as well as the outcomes, of chILD in a Turkish cohort classified according to the chILD-EU framework.

**Materials and methods:**

We retrospectively reviewed the medical records of 84 pediatric patients diagnosed with chILD between 2017 and 2024 at a tertiary referral center in Türkiye. Patients were categorized according to the chILD-EU classification. Clinical variables, imaging findings, genetic analyses, pulmonary function test results, and Fan severity scores were systematically assessed. Logistic regression analysis was performed to identify independent predictors of clinical instability.

**Results:**

The median age at diagnosis was 6.0 years (IQR: 1.1–12.9). Surfactant dysfunction disorders (A4) and immune- or environmental-related diseases (B2) were the most frequently identified subtypes. Hypoxia was observed in 36 of 84 patients (42.8%) and emerged as the strongest independent predictor of clinical instability (OR: 8.5; 95% CI: 2.2–33.0; p = 0.002). Pathogenic or likely pathogenic variants were identified in 18 of 84 patients (21.4%); among variant-positive cases, *ABCA3* was the most frequently affected gene (3 of 18; 16.7%). Chest computed tomography was available in 82 of 84 patients, with ground-glass opacities being the most common finding, observed in 51 of 82 patients (62.2%). A decrease of at least one point in the Fan severity score was observed in 42 of 84 patients (p < 0.001). Mortality was 12 of 84 patients (14.3%) after a median follow-up of 3.2 years (range: 1.2–4).

**Conclusion:**

This study presents one of the largest single-center pediatric chILD cohorts reported from Türkiye. It highlights the prognostic relevance of baseline hypoxia and underscores the importance of comprehensive radiological and genetic assessment in the management of chILD.

## 1. Introduction

Interstitial lung diseases (ILDs) represent a heterogeneous group of rare and complex respiratory disorders affecting the interstitium, alveoli, distal small airways, and/or terminal bronchioles in infants and children [1]. These disorders are complicated by diverse underlying mechanisms initiated by primary injurious processes. Overall, the identification and characterization of ILDs in children, particularly in infants, remain a major clinical challenge. A stringent diagnostic process is required to investigate correlations between pathological markers and patient phenotypes, thereby advancing the understanding and classification of these diseases [2].

However, reliable epidemiological estimates are lacking; the prevalence is likely <1 per 100,000 in childhood, compared with 60–80 per 100,000 in adults [3], and the average hospital in Europe is not expected to encounter more than five cases per year [4]. The prevalence and incidence of ILDs are likely underestimated because the disease spectrum is broad and clinical manifestations are often nonspecific. Consequently, pediatric ILDs appear to be approximately 10 times rarer than those in adults, exhibit distinct etiologies, and may present with extreme severity [5,6].

Due to its rare occurrence and broad clinical spectrum, there has been no consensus regarding disease terminology, classification, treatment, or follow-up in childhood interstitial lung diseases (chILD) [7]. The classification of chILD is based on etiology and pathology and consists of two main groups: A–diffuse parenchymal lung disease (A-DPLD), comprising disorders that manifest primarily in infancy, and B–diffuse parenchymal lung disease (B-DPLD), which includes disorders occurring at all ages [8]. When chILD is suspected, further evaluation by experienced clinicians, geneticists, pathologists, and radiologists is recommended. Despite comprehensive assessment, approximately 6%–12% of chILD cases remain unexplained or of unidentified etiology [6,9].

Overall, clinical outcomes in chILD are highly variable. Outcomes are strongly influenced by underlying etiology, the patient care environment, and individual responses to treatment. Oxygen requirement, respiratory function parameters, and the presence of pulmonary hypertension are among the most important clinical markers for predicting prognosis in chILD [10]. The management of chILD represents a significant challenge. Current treatment strategies are largely based on expert opinion, with few randomized controlled trials available to guide management. In most cases, treatment remains supportive rather than curative [2,11,12].

This study aimed to characterize the clinical, radiological, and genetic features, as well as outcomes, of chILD in a Turkish cohort using the chILD –EU classification. This study is expected to provide insights into improving patient care and supporting future research by examining prognostic outcomes in patients diagnosed and classified according to the latest chILD-EU framework at a tertiary referral center.

## 2. Materials and methods

### 2.1. Study design and participants

This single-center retrospective cohort study included patients with chILD who were diagnosed and followed up at our tertiary referral center between March 2017 and March 2024.

Electronic medical records of the patients were reviewed. Demographic, clinical, and radiologic characteristics; personal and family history; treatment data; bronchoscopy findings; genetic test results; and lung biopsy findings were retrospectively analyzed.

### 2.2. Definitions and procedures

**Figure 1:**
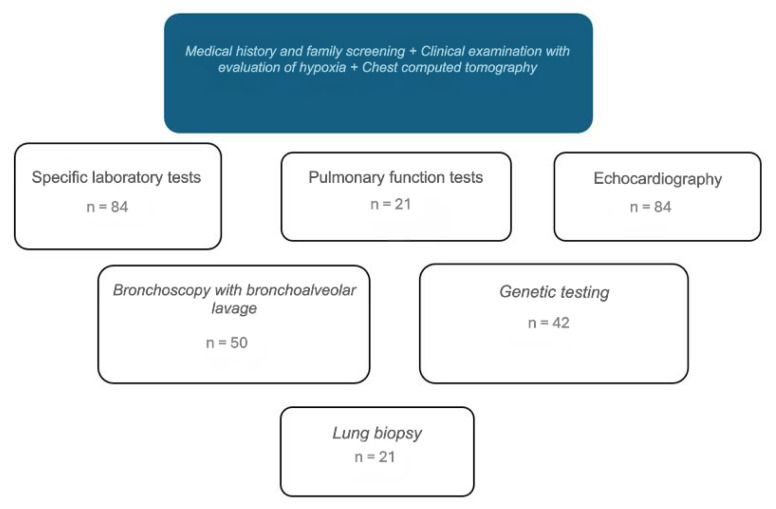
Investigations used in the diagnostic approach to childhood interstitial lung disease at our center.

**Figure 2:**
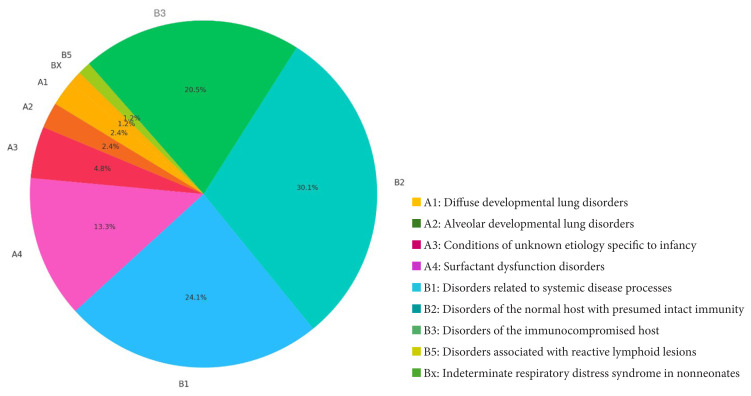
Distribution of chILD subcategories.

Cases were defined according to the chILD syndrome criteria, requiring the presence of at least three of the following features: (1) respiratory symptoms (cough, rapid and/or difficult breathing, exercise intolerance); (2) respiratory signs (tachypnea, adventitious sounds, retractions, digital clubbing, failure to thrive, or respiratory failure); (3) hypoxemia or low pulse oxygen saturation; and (4) diffuse abnormalities on chest radiography or computed tomography (CT). Patients with underlying conditions such as lung infections, cystic fibrosis, bronchopulmonary dysplasia, congenital heart disease, and primary ciliary dyskinesia were excluded from the study. The final diagnosis was established based on the clinical presentation, CT scan findings, and, when applicable, results from lung biopsy or genetic tests. Figure 1 illustrates the diagnostic scheme used at our center. Diseases were categorized according to the chILD-EU working group classification [9,11,12]. The two principal categories of DPLD were disorders occurring primarily in infancy (A-DPLD) and those occurring at any age (B-DPLD). Patients were further classified into subcategories of A-DPLD and B-DPLD. These subcategories are defined in Figure 2. Hypoxia was defined as an arterial oxygen partial pressure (PaO_2_) of 60 mmHg (8 kPa) or an oxygen saturation (SaO_2_) of ≤94% on room air [13]. Chest CT imaging was available in 82 of 84 patients; high-resolution computed tomography (HRCT) was performed in 11 patients, and standard chest CT was performed in the remaining 71 patients. All chest CT scans were performed using a GE Revolution 64-slice scanner (GE Healthcare, Chicago, IL, USA) at 80–100 kV, with a slice thickness of <2 mm, a 512 × 512 matrix, and a 0° gantry angle. HRCT scans were acquired at 120–140 kV with a slice thickness of 1 mm. Younger children were scanned in the supine position during quiet breathing, whereas older children performed breath-holding as instructed. CT images were evaluated by the pediatric radiology team at our center, and all findings were also evaluated by the pediatric pulmonology team at the time of diagnosis. Diagnoses were established based on specific CT features, including ground-glass opacities; nodules (size and characteristics); interlobular septal thickening; honeycombing; parenchymal distortion; emphysematous changes; traction bronchiectasis; and areas of consolidation [14].

Z-scores for forced expiratory volume in 1 s (FEV_1_), forced vital capacity (FVC), and the FEV_1_/FVC ratio were calculated according to the Global Lung Function Initiative (GLI) 2012 multiethnic spirometry reference values [15].

Genetic analyses were performed sequentially, initially targeting surfactant-related genes (SFTPB, SFTPC, ABCA3, and NKX2-1) and pulmonary alveolar proteinosis–related genes (CSF2RA and CSF2RB), followed by next-generation sequencing and whole-exome sequencing. All genetic analyses were conducted in the genetic laboratory of our center. Genetic variant interpretation was independently reviewed by a certified genetic counselor. Variants were interpreted according to the American College of Medical Genetics and Genomics (ACMG) five-tier framework (pathogenic, likely pathogenic, variant of uncertain significance, likely benign, or benign) [16]. Variants that lacked sufficient evidence for either pathogenicity or benignity were classified as variants of uncertain significance (VUS). Although these VUS did not meet strict pathogenicity criteria, the corresponding patients exhibited clinical and radiological findings consistent with reported gene-related disease phenotypes, suggesting a possible association.

Pharmacological management primarily involves antiinflammatory and immunosuppressive agents, with systemic corticosteroids used as first-line therapy. Oral prednisolone (1–2 mg/kg/day) or pulsed methylprednisolone (30 mg/kg/day for 3 consecutive days per month) was administered depending on disease severity, followed by dose tapering once disease control was achieved [11]. Hydroxychloroquine (6–10 mg/kg/day) was used as an alternative, particularly in cases with increased collagen deposition suggestive of prefibrotic changes, whereas corticosteroids were favored in the presence of marked inflammatory disease [17]. Macrolides with immunomodulatory effects were also added when appropriate [18].

In steroid- or hydroxychloroquine-refractory cases, additional immunosuppressive agents (azathioprine, cyclophosphamide, cyclosporine, and mycophenolate mofetil [MMF]) were used, particularly in chronic or relapsing disease. Antifibrotic agents (pirfenidone and nintedanib) were considered in cases of progressive fibrosis, and the fluticasone–azithromycin–montelukast (FAM) regimen was used as adjunctive therapy in bronchiolitis obliterans syndrome following allogeneic hematopoietic stem cell transplantation or in milder interstitial lung disease with predominant airway inflammation.

**Figure 3:**
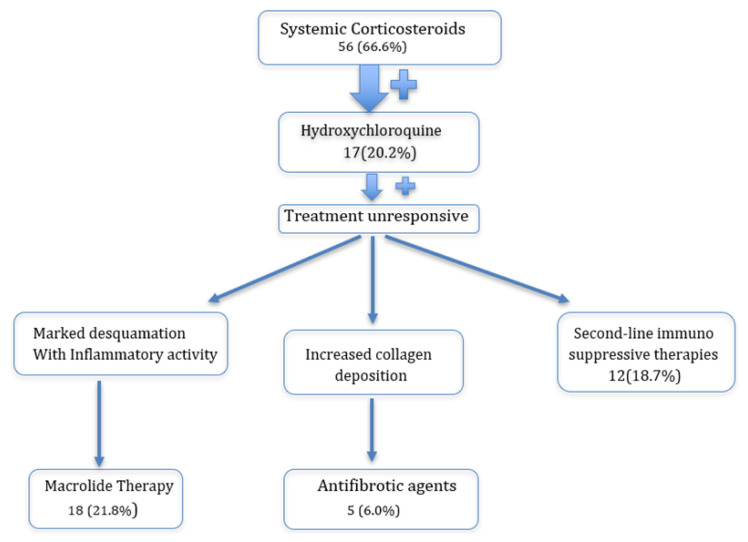
Flowchart of the treatment steps used at our center.

All treatments required routine safety monitoring, including liver and renal function tests, complete blood counts, ophthalmologic evaluation for hydroxychloroquine, growth and metabolic assessments for systemic corticosteroids, and liver enzyme surveillance during antifibrotic therapy [19,20]. The flowchart illustrating the treatment steps used at our center is shown in Figure 3.

The Fan disease severity score, based on symptoms, degree of hypoxia, and the presence of pulmonary hypertension, was graded as follows: (1) asymptomatic; (2) symptomatic with normal room air oxygen saturation under all conditions; (3) symptomatic with normal resting room air saturation but abnormal saturation during sleep or exercise (<90%); (4) symptomatic with abnormal resting room air saturation (<90%); and (5) symptomatic with pulmonary hypertension [21]. The investigator categorized patients’ clinical outcomes at the last follow-up visit according to Fan’s disease severity score; patients with a score of ≥3 were defined as clinically unstable, whereas those with a score of ≤2 were considered clinically stable.

A comparative analysis was undertaken to examine demographic, clinical, and radiologic characteristics, including age, sex, parental consanguinity, history of intensive care unit admission, chest CT/HRCT findings, pulmonary function test results within the preceding year, histopathological and bronchoalveolar lavage findings, time from symptom onset to diagnosis, treatments, and outcomes in patients with A-DPLD and B-DPLD.

### 2.3. Statistical analysis

Statistical analyses were performed using SPSS version 22 (IBM Corp., Armonk, NY, USA). The normality of continuous variables was assessed using the Shapiro–Wilk test. Normally distributed continuous variables were analyzed using Student’s t-test and are presented as mean ± standard deviation. Continuous variables that were not normally distributed were analyzed using the Mann–Whitney U test and are presented as the median (first–third quartiles [Q1–Q3]). Categorical variables are presented as frequencies and percentages. The chi-square (χ^2^) test, with or without continuity correction, or Fisher’s exact test was used, as appropriate. A p value of <0.05 was considered statistically significant. A logistic regression model was constructed to evaluate covariates with clinical and/or statistical significance.

## 3. Results

### 3.1. Patient characteristics

This study included 84 patients with DPLD diagnosed based on clinical, laboratory, bronchoscopy, radiological, and histopathological findings, as well as genetic analyses. Data were obtained between March 2017 and March 2024. For each patient, age and outcome at the most recent visit were recorded. The median age at diagnosis was 6.0 years (range: 1.1–12.9 years). Overall, 56% of the patients were male, and the current median age was 10.6 years (range: 4.0–16.8). Table 1 summarizes the demographic characteristics, clinical features, and examination findings of the patients. The most common symptoms and signs on admission were dyspnea (n = 54, 64.2%) and lower respiratory tract infection (n = 28, 33.3%). Hypoxia (n = 36, 42.8%) and rales (n = 31, 36.9%) were the most common examination findings. The median time from symptom onset to diagnosis was 2.7 months (range: 1.0–12.0). The median follow-up duration was 3.2 years (range: 1.2–4).

A total of 82 patients (97.6%) underwent at least one chest CT or HRCT examination. The most common chest CT/HRCT finding was ground-glass opacities, observed in 51 patients (62.2%). The chest CT/HRCT findings are summarized in Table 1.

Flexible fiberoptic bronchoscopy was performed in 50 patients (59.5%). Bronchoalveolar lavage was diagnostic in patients with pulmonary hemosiderosis, hypereosinophilic syndrome, lipid storage disease, and pulmonary alveolar proteinosis.

Genetic testing was performed in 42 of 84 patients (50.0%), and pathogenic or likely pathogenic variants were identified in 18 of these 42 patients (42.9%), corresponding to 21.4% of the overall cohort (18 of 84). Mutations associated with surfactant metabolism were identified in seven patients (8.3%). Pathogenic or likely pathogenic variants associated with interstitial lung disease were identified in ABCA3 (n = 3), NKX2-1 (n = 2), SLC34A2 (n = 2), AKR1D1 (n = 1), CCR2 (n = 1), COPA (n = 1), DOCK8 (n = 1), FARSB (n = 1), FOXF1 (n = 1), ITGA3 (n = 1), MARS1 (n = 1), and TBX4 (n = 1).

Lung biopsy was performed in 21 patients and was diagnostic in 17 of these patients (81.0%), corresponding to 20.2% of the overall cohort (17 of 84). Biopsy showed fibrosis, chronic inflammation, and pleuroparenchymal fibrosis.

Pulmonary function tests (PFTs) were available for 21 of 84 patients (25.0%), including two patients classified as A-DPLD. Therefore, the values are presented descriptively and should not be generalized to the entire cohort. The results are consistent with a predominantly restrictive pattern within the tested subset. The median Z-scores were −2.22 (range: −2.87 to −1.08) for FEV_1_, −1.84 (range: −2.99 to −0.95) for FVC, and 0.71 (range: −0.22 to 1.44) for the FEV_1_/FVC ratio.

Systemic corticosteroid therapy was administered to 56 patients (66.6%). An overview of treatment modalities is provided in Table 1.

### 3.2. Comparison between A-DPLD and B-DPLD groups

**Figure 4:**
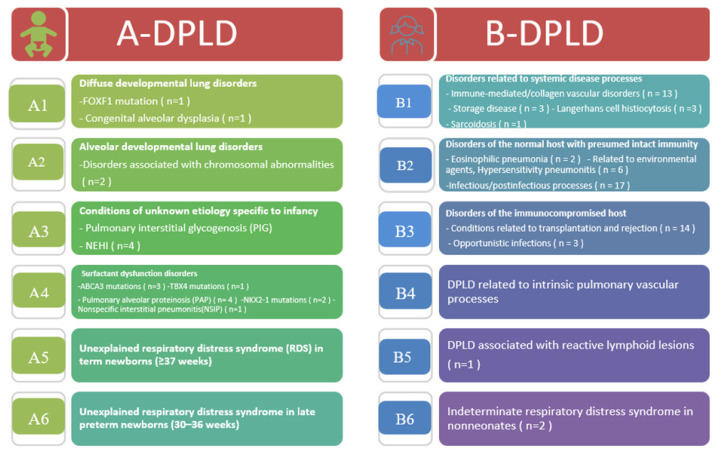
Subcategories and diagnoses of diffuse parenchymal lung disease (DPLD). A-DPLD: A-diffuse parenchymal lung disease; disorders manifesting primarily in infancy. B-DPLD: B-diffuse parenchymal lung disease; disorders occurring at all ages. NEHI: neuroendocrine cell hyperplasia of infancy. ABCA3: ATP binding cassette subfamily A member 3. COPA: coatomer protein complex subunit alpha. RDS: respiratory distress syndrome. FOXF1: forkhead box F1. TBX4: T-box transcription factor 4. NKX2-1: NK2 homeobox 1.

Within the study cohort, 65 patients (77.3%) were classified as B-DPLD, with B2 representing the predominant subcategory (30.1%). The most prevalent subcategory within the A-DPLD group was A4, encompassing surfactant dysfunction disorders. Figures 2 and 4 illustrate the distribution of diagnostic groups and chILD subcategories within the study cohort.

There was no significant difference between groups A and B in terms of the frequency of dyspnea. The frequency of intensive care unit (ICU) hospitalization was higher in the A-DPLD group (p < 0.001), whereas the frequency of rales detection was higher in the B-DPLD group (p = 0.034).

No significant differences were observed between the A-DPLD and B-DPLD groups in the frequency of ground-glass opacities (p = 0.984), mosaic attenuation (p = 1.0), or interlobular septal thickening (p = 0.169).

There was no significant difference in treatment distribution between the A-DPLD and B-DPLD groups.

**Figure 5:**
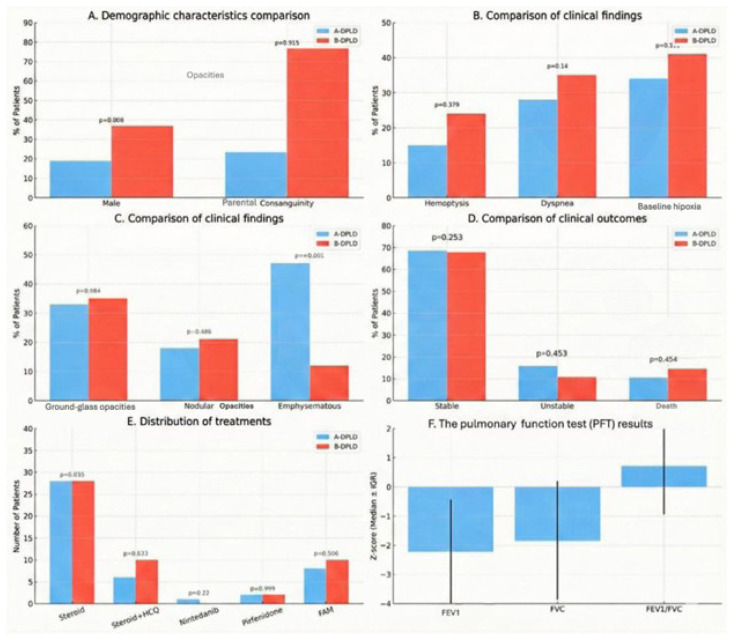
Comparison of chILD subcategories according to clinical findings, treatment, and outcomes. A. Demographic comparison of subcategories: 16 patients (19.0%) in the A-DPLD group and 31 patients (36.9%) in the B-DPLD group were male (p = 0.008). Parental consanguinity was present in 30 patients (35.7%), including seven patients (23.3%) in the A-DPLD group and 23 patients (76.7%) in the B-DPLD group (p = 0.915). B. Comparison of clinical findings: No significant differences were observed between the A-DPLD and B-DPLD groups in the frequency of hemoptysis (p = 0.329), dyspnea (p = 0.140), or baseline hypoxia (p = 0.132). C. Comparison of radiological findings: No significant differences were observed between the groups in the frequency of ground-glass opacities (p = 0.984) or nodular opacities (p = 0.486). Emphysematous changes were significantly more frequent in the A-DPLD group (p < 0.001). D. Comparison of clinical outcomes: During follow-up, 56 patients (67.8%) were clinically stable, including 13 patients (68.4%) in the A-DPLD group and 44 patients (67.7%) in the B-DPLD group (p = 0.253). A total of 10 patients (11.9%) were classified as unstable, comprising three patients (15.8%) in the A-DPLD group and seven patients (10.8%) in the B-DPLD group (p = 0.453). During follow-up, a total of 12 patients (14.3%) died, including two patients (10.5%) in the A-DPLD group and 10 patients (14.4%) in the B-DPLD group (p = 0.454). A total of five patients (5.9%) were lost to follow-up. E. Comparison of treatments: Overall, 56 patients (66.6%) received systemic corticosteroids alone. In addition to corticosteroids, hydroxychloroquine was administered to 17 patients (20.2%), antifibrotic agents were used in five patients (nintedanib in one patient [1.1%] and pirfenidone in four patients [4.7%]), and the fluticasone–azithromycin–montelukast (FAM) regimen was used in 18 patients (21.4%). No significant differences were observed in treatment distribution between the A-DPLD and B-DPLD groups (p = 0.635, p = 0.633, p = 0.220, p = 0.999, and p = 0.506, respectively). F. Pulmonary function test results: Pulmonary function tests were available for a limited subset of patients. The median (Q1–Q3) Z-scores were −2.2 (−2.9 to −1.1) for FEV_1_, −1.8 (−3.0 to −0.9) for FVC, and 0.71 (−0.2 to 1.4) for the FEV_1_/FVC ratio.

Figure 5 illustrates comparisons among chILD subcategories based on clinical findings, treatment modalities, and outcomes.

The median Fan score at diagnosis was 3.00 (IQR: 2–4) and decreased significantly to 2.00 (IQR: 2–3) at follow-up (p < 0.001). Patients were classified into two groups based on their most recent clinical status: unstable or deceased patients and clinically stable patients. There was no significant difference in stability status between males and females (p = 1.0).

Similarly, the diagnostic category was not associated with stability status. In the stable group, 8 of 59 patients (13.1%) were classified as A-DPLD, compared with 2 of 25 patients (9.5%) in the unstable group. Similarly, 34 of 59 stable patients (57.1%) and 5 of 25 unstable patients (20.2%) were categorized as B-DPLD, with no significant difference between groups (p = 0.293).

At baseline, dyspnea (p < 0.001) and rales (p = 0.026) were significantly more frequent in the stable group, whereas hypoxia (p < 0.001), higher initial Fan scores (p < 0.001), the presence of interlobular septal thickening on chest CT (p = 0.013), and the use of hydroxychloroquine (p < 0.001) and MMF (p = 0.022) were significantly more frequent in the unstable group. A comparison between the onset groups is shown in Table 2. Associations observed for treatment variables, such as hydroxychloroquine and mycophenolate mofetil, likely reflect indication bias, as these agents were preferentially prescribed to patients with more severe or progressive disease. In the A-DPLD group, eight patients (42.1%) were classified as unstable, compared with 17 patients (26.1%) in the B-DPLD group, with no significant difference between groups (p = 0.253). In multivariable analysis (Table 3), hypoxia at presentation remained the only independent predictor of poor outcome (OR: 8.5; 95% CI: 2.21–33.0; AUC: 0.87; p = 0.002). Clinical improvement, defined as a reduction of at least one point in the Fan score, was observed in 42 patients (50%). Overall mortality was observed in 12 of 84 patients (14.3%) after a median follow-up of 3.2 years (range: 1.2–4).

## 4. Discussion

This study presents one of the most comprehensive characterizations of childhood interstitial lung diseases (chILD) in Türkiye by integrating genetic, radiological, and clinical data to identify key prognostic factors. Among the evaluated factors, baseline hypoxia emerged as the only independent predictor of adverse outcomes, underscoring its clinical relevance as an early marker of disease severity. Through detailed clinical analyses in a large single-center cohort, this study contributes to the existing literature on chILD follow-up and outcomes. In addition, disease subgroups were examined, providing a comprehensive characterization of children diagnosed and classified according to the chILD-EU criteria [5,12,22,23].

Childhood interstitial lung disease (chILD) presents unique diagnostic challenges owing to nonspecific symptoms and a broad clinical spectrum [21,24,25]. This study highlights hypoxia, recurrent infections, and rales as the most common clinical manifestations, consistent with prior reports. However, wide variability in symptom severity, ranging from mild hypoxia to severe respiratory distress, frequently results in delayed diagnosis [2,21,24,26,27]. A particularly notable finding is this cohort is the high prevalence of parental consanguinity (35.7%) among affected families, underscoring a strong genetic contribution to chILD, particularly in conditions related to surfactant metabolism and immune deficiencies [2,3,28]. The median time from symptom onset to diagnosis was 2.7 months, representing an improvement compared with international cohorts but still emphasizing the need for heightened clinical suspicion. Early diagnosis appears to be particularly critical for preventing disease progression in patients with A-DPLD.

The predominance of the A4 (surfactant dysfunction disorders) and B2 (environmental-related diseases) subcategories in this cohort distinguishes the distribution of chILD in Türkiye from global trends [6,8,22]. These differences may reflect regional and environmental influences, disparities in healthcare access and diagnostic infrastructure, as well as variations in genetic and environmental exposures. These findings highlight the importance of local data for improving the understanding of chILD and for adapting diagnostic and management strategies accordingly.

A recent registry from the Children’s Interstitial and Diffuse Lung Disease Research Network (chILDRN) in the United States, including 683 patients, reported neuroendocrine cell hyperplasia of infancy (NEHI) as the most prevalent diagnosis (23%), followed by chILD associated with connective tissue or immune-mediated diseases (16.5%) [25]. In the present cohort, the prevalence of NEHI was 21%. In a multicenter study by Deutsch et al. that included 187 children younger than 2 years of age with DPLD, surfactant protein C (SFTPC) mutations were identified in seven patients, and ABCA3 mutations were identified in six patients [6]. In contrast, the present study identified surfactant metabolism disorders as the most common genetic basis, although no SFTPC mutations were detected. ABCA3 mutations were identified in three patients. The higher frequency of ICU admissions observed in infant-onset chILD compared with older cases suggests a potential association between pulmonary fibrosis and genetic mutations affecting lung development [29]. These findings underscore the important role of genetic mutations, particularly those related to surfactant metabolism, in disease pathogenesis and severity, thereby reinforcing the potential value of early genetic screening and targeted interventions in this vulnerable population.

Recent advances in molecular genetics have prompted expert chILD groups, such as the American Thoracic Society and the Clinical Research Collaboration of the European Respiratory Society, to reevaluate the indications for lung biopsy. While both lung biopsy and genetic testing are considered in diagnostic workups, current recommendations suggest that genetic analysis should precede lung biopsy [24,26]. In line with these recommendations, the present study emphasizes the significance of genetic testing, which identified pathogenic or likely pathogenic variants in 21.4% of patients, most frequently involving ABCA3. These findings are consistent with the current literature emphasizing the importance of early genetic screening to improve diagnostic accuracy [12].

Among patients who underwent lung biopsy, a definitive diagnosis was achieved in 81% of cases [25]. However, most cases were diagnosed without invasive procedures through multidisciplinary assessments and genetic analysis. Surfactant dysfunction disorders (A4 subgroup) and NEHI were frequently diagnosed based solely on clinical, radiological, and genetic data. These findings reflect global trends that favor genetic testing as an initial diagnostic step, with lung biopsy reserved for cases with inconclusive findings.

Chest CT and HRCT, in conjunction with clinical evaluation, remain critical diagnostic tools in chILD [19,30]. Ground-glass opacities were observed in 60.7% of patients, whereas emphysematous changes were predominantly associated with A-DPLD. Interestingly, although mosaic attenuation and interlobular septal thickening were common radiological features across all age groups [16,24–26], emphysematous changes showed potential age- or etiology-related distinctions in chILD pathophysiology. This divergence from the existing literature underscores the need for further studies to clarify age-related imaging patterns and refine diagnostic criteria [23,31,32].

Pulmonary function tests (PFTs) have limited utility for diagnostic purposes in younger patients because of reliability concerns [33]. Although pulmonary function tests (PFTs) are generally more reliable in older children, they remain valuable for longitudinal assessment of disease severity, monitoring disease progression, and evaluating treatment response [34,35]. PFT data were available for 21 of 84 patients (25.0%). The results are consistent with a predominantly restrictive pattern within the tested subset.

This study reaffirms the central role of systemic corticosteroids in the management of chILD, which were used in 66.6% of patients. Additional therapies, including hydroxychloroquine, the FAM regimen, and antifibrotic agents, were tailored to patients’ clinical and genetic profiles [11,17,18]. The greater use of hydroxychloroquine and MMF in the unstable group likely reflects indication bias rather than treatment-related harm. Immunosuppressive agents appeared to be particularly beneficial in immune-related disorders; for example, patients with coatomer protein complex subunit alpha (COPA) mutations showed favorable responses to cyclophosphamide following corticosteroid failure. These findings highlight the importance of personalized treatment strategies informed by genetic and clinical data in the management of chILD [36].

Despite the widespread use of systemic corticosteroids, concerns regarding their long-term effects underscore the need for alternative therapeutic strategies [20,26,32]. Although currently used sparingly, antifibrotic agents such as nintedanib and pirfenidone show potential for future therapeutic use. The findings emphasize the need for standardized treatment protocols to reduce variability and improve clinical outcomes.

Clinical outcomes vary considerably, and fatalities continue to be reported. Although the observed mortality rate of 14.3% was lower than that reported in previous studies [6,21,37], it continues to reflect the severity of chILD. In a recent prospective cohort study including 127 children, Cunningham et al. reported developmental or surfactant disorders, baseline age younger than 6 months, and baseline SpO2 below 94% as factors associated with poor prognosis. In the present study, baseline hypoxia and the extent of initial fibrosis were associated with clinical instability [10]. Although prior research has suggested that infancy-onset cases have a poorer prognosis [6,38], the present study found no significant difference in outcomes between the A-DPLD and B-DPLD groups. This finding may be attributable to the absence of a distinct subgroup for surfactant protein C deficiency and to the study center serving as a referral center for bone marrow transplantation.

Longitudinal assessment of disease severity is essential for preventing disease progression. The Fan score is a valuable tool in this regard. The high initial Fan scores observed in patients in the unstable group are consistent with findings reported in the literature [21]. Clinical improvement was reported in 74% of pediatric patients with chILD in a cohort of 185 individuals [39], whereas a 50% improvement in Fan scores was observed in the present cohort following treatment. Analysis of Fan scores at diagnosis and follow-up demonstrated a significant overall decline, indicating improvement in clinical condition and response to therapy. No significant association was observed between final Fan scores and A-DPLD or B-DPLD classification, suggesting that the score reflects overall disease severity rather than specific subcategory classification. Consistent with prior studies, hypoxia and pulmonary hypertension remain key indicators of clinical severity in chILD [21]. These findings indicate that hypoxia at presentation, independent of pulmonary hypertension, is the most valuable predictor of progression to an unstable clinical state. In conclusion, although the Fan score is a useful clinical tool, it has limitations in distinguishing disease subcategories and predicting long-term outcomes.

## 5. Limitations

Despite being one of the most comprehensive single-center analyses of childhood interstitial lung disease (chILD) in Türkiye, this study has several limitations. As a single-center study, the findings may not be generalizable to the broader population. Multicenter studies are needed to better assess regional and genetic variability. Not all patients underwent genetic testing, which may have resulted in underrepresentation of specific genetic subtypes and missed mutations, particularly in diagnostically challenging cases. Retrospective data collection increases the risk of missing or inaccurately recorded information, particularly for parameters such as the time from symptom onset to diagnosis. Furthermore, the study lacks long-term outcome data, which limits the evaluation of treatment effectiveness and the assessment of complications or mortality. The small number of patients in certain subgroups, including A-DPLD and B-DPLD, limited the statistical power and may have prevented the detection of meaningful differences.

## 6. Conclusion

This study provides a comprehensive characterization of chILD and identifies baseline hypoxia as the only significant predictor of clinical instability. These findings highlight the importance of multidisciplinary evaluation in the follow-up of patients with hypoxia. In addition, the combined use of genetic testing with clinical and radiological findings was found to be useful for diagnosis.

By presenting one of the largest single-center chILD cohorts in Türkiye classified according to the chILD-EU criteria, this study provides valuable regional data that contribute to the global understanding of this rare and heterogeneous disease group.

## Figures and Tables

**Table 1:** Demographic and clinical characteristics of patients with chILD.

	All(84)	A-DPLD (19)	B-DPLD (65)	p
**Demographic findings**				
• Male sex, n (%)	47 (56.0)	16 (19.0)	31 (36.9)	0.008
• Age at last visit, years, median (Q1–Q3)	10.6 (4.0–16.8)	3.9 (2.9–7.3)	12.2 (6.9–17.4)	<0.001
• Age at diagnosis, years, median (Q1–Q3)	6.0 (1.1–12.9)	0.5 (0.0–2.0)	7.9 (2.7–14.0)	<0.001
•Time from symptom onset to diagnosis, months, median (Q1–Q3)	2.7 (1.0–12)	4 (2.2–14.5)	6 (1–16)	0.663
• Parental consanguinity, n (%)	30 (35.7)	7 (23.3)	23 (76.7)	0.915
• History of NICU/PICU admission, n (%)	23 (27.5)	13 (56.5)	10 (43.5)	<0.001
**Clinical findings, n (%)**				
• Hypoxia	36 (42.8)	11 (30.6)	25 (69.4)	0.132
• Dyspnea	54 (64.2)	15 (78.9)	39 (60)	0.213
• Recurrent lower respiratory tract infections	28 (33.3)	9 (32.1)	19 (67.9)	0.140
• Hemoptysis	6 (7.1)	0	6 (9.2)	0.329
**Pulmonary auscultation findings, n (%)**				
• Rales	31 (36.9)	3 (3.5)	28 (33.3)	0.034
• Rhonchi	16 (19.0)	2 (12.5)	14 (87.5)	0.506
**Tomography findings, n (%)** †				
• Ground-glass opacities	51 (62.2)	11 (57.9)	40 (61.5)	0.984
• Interlobular septal thickening	19 (23.2)	7 (36.8)	12 (18.5)	0.169
• Mosaic attenuation pattern	21 (25.6)	5 (26.3)	16 (24.6)	0.999
• Nodular opacities	5 (6.1)	0	5 (7.7)	0.486
• Emphysematous changes	13 (15.9)	8 (42.1)	5 (7.7)	0.001
**Pulmonary function test findings, n = 21**				
• Forced expiratory volume in 1 s (FEV_1_), Z-score, median (Q1–Q3)	−2.2 (−2.9–1.1)	−2.9 (0.07)	−2.0 (1.6)	0.013
• Forced vital capacity (FVC), Z-score, median (Q1–Q3)	−1.8 (−3.0–0.9)	−3.1 (0.07)	−1.5 (1.9)	0.001
• FEV_1_/FVC ratio, Z-score, median (Q1–Q3)	0.7 (−0.2–1.4)	1.1 (0.65)	0.7 (1.9)	0.352
**Treatment, n (%)**				
• Systemic corticosteroids	56 (66.6)	7 (36.8)	49 (75.3)	0.635
• Hydroxychloroquine	17 (20.2)	4 (21.0)	13 (20)	0.633
• Fluticasone–azithromycin–montelukast (FAM) regimen	18 (21.4)	1 (5.2)	17 (20.2)	0.566
• Antifibrotic agents (nintedanib, pirfenidone)	5 (5.9)	1 (5.2)	4 (6.15)	0.220
•Immunosuppressive therapies (cyclosporine, mycophenolate mofetil)	12 (18.2)	2 (10.5)	10 (15.3)	0.633
**Outcome, n (%)**				
• Stable	57 (67.8)	13 (68.4)	44 (67.7)	0.253
• Unstable	10 (11.9)	3 (15.8)	7 (10.8)	0.453
• Death	12 (14.3)	2 (10.5)	10 (14.4)	0.454
• Unknown outcome	5 (5.9)	1 (5.3)	4 (6.1)	0.53

NICU: neonatal intensive care unit; PICU: pediatric intensive care unit; DPLD: diffuse parenchymal lung disease.

† Chest CT or HRCT findings are reported for patients with available imaging (n = 82).

Percentages in the “All patients” column were calculated using n = 82 as the denominator, whereas percentages in the A-DPLD and B-DPLD columns were calculated using group-specific denominators (n = 19 and n = 65, respectively).

**Table 2:** Comparison of clinical outcomes between groups.

Variable	Stable, n (%)	Unstable, n (%)	p
Age at last visit (years), mean	10.1 ± 6.7	12.1 ± 6.9	0.213
Age at diagnosis (years), mean	6.3 ± 5.8	8.3 ± 6.1	0.164
Initial Fan score at diagnosis, mean	2,7 ± 0.9	4.3 ± 0.7	**<0.001**
Time from symptom onset to diagnosis (months), median (Q1–Q3)	3.0 (1–10.5)	6 (0.6–16)	0.834
History of recurrent lower respiratory tract infections	18 (30.5%)	9 (36.0%)	0.720
NICU history	18 (30.5%)	5 (20.0%)	0.546
**Signs and symptoms at first visit**			
Rales	17 (28.8%)	14 (56.0%)	**0.035**
Rhonchi	12 (20.3%)	4 (16.0%)	0.874
Hypoxia	16 (27.1%)	20 (80.0%)	**<0.001**
Pulmonary hypertension	11 (14.7%)	5 (6.7%)	1.000
**Chest CT findings at the time of diagnosis**			
Ground-glass opacities	34 (57.6%)	17 (68.0%)	0.518
Interlobular septal thickening	9 (15.3%)	10 (40.0%)	**0.028**
Mosaic attenuation	18 (30.5%)	3 (12.0%)	0.130
Emphysematous changes	9 (15.3%)	4 (16.0%)	1.000
Traction bronchiectasis	0 (0.0%)	4 (16.0%)	0.010
**Treatments**			
Hydroxychloroquine	5 (8.5%)	11 (44.0%)	**<0.001**
Fluticasone–azithromycin–montelukast regimen	23 (41.1%)	10 (35.7%)	0.636
Mycophenolate mofetil	2 (3.4%)	5 (20.0%)	**0.037**
Pirfenidone	0 (0.0%)	4 (16.0%)	**0.010**

NICU: neonatal intensive care unit; RLTI: recurrent lower respiratory tract infections; MMF: mycophenolate mofetil; FAM: fluticasone–azithromycin–montelukast.

**Table 3:** Predictors of clinical outcomes based on statistical analysis.

Variable	B (Estimate)	OR (95% CI)	p
Baseline hypoxia at first visit	2.14	8.5 (2.2–33.0)	**0.002**
Male sex	0.41	1.5 (0.4–5.8)	0.547
Rales at initial presentation	0.74	2.0 (0.5–8.0)	0.283
Interlobular septal thickening on chest CT	−3.31	2.7 (0.7–11.3)	0.157
Treatment with hydroxychloroquine	1.21	3.3 (0.7–16.4)	0.138
Treatment with mycophenolate mofetil (MMF)	1.03	2.8 (0.3–27.9)	0.385

CT: computed tomography.
